# Impact of a vancomycin-resistant *Enterococcus* (VRE) screening result on appropriateness of antibiotic therapy

**DOI:** 10.1017/ash.2021.215

**Published:** 2021-11-03

**Authors:** Jenna L. Reynolds, Raelene E. Trudeau, Maria Teresa Seville, Lynn Chan

**Affiliations:** 1 Department of Pharmacy, Mayo Clinic Hospital, Phoenix, Arizona; 2 Department of Pharmacy, University of Texas Southwestern Medical Center, Dallas, Texas; 3 Division of Infectious Diseases, Mayo Clinic Hospital, Phoenix, Arizona

## Abstract

**Objective:**

Vancomycin-resistant *Enterococcus* (VRE) infections have been associated with increased mortality and poor outcomes. VRE screening has been used to identify colonized patients to prevent transmission; however, little is known about the utility of screening results to guide antibiotic therapy.

**Design and setting:**

A retrospective review was performed at a tertiary-care center between June 1, 2015, and May 31, 2018.

**Patients:**

All patients who underwent VRE polymerase chain reaction assay (PCR) screening and had a bacterial culture from 7 days before to 90 days after the screening test were included. In total, 1,374 patients who had a VRE screening test met inclusion criteria.

**Methods:**

Sensitivity, specificity, and positive and negative predictive values of VRE screening for VRE infection were calculated. The appropriateness of the antibiotic therapy for each patient based on screening results was also assessed.

**Results:**

We detected no difference in the appropriateness of antibiotic therapy between patients with a positive screen and those with a negative screen (59.3% vs 61.0%; *P* = .8657). The VRE PCR demonstrated 54% sensitivity, 89% specificity, a positive predictive value (PPV) of 13% and a negative predictive value (NPV) of 98%.

**Conclusions:**

The high NPV and specificity indicate that patients with a negative VRE screening results may not require empiric antibiotic coverage for VRE. Although VRE screening may have utility to detect colonization in high-risk patients, a positive VRE screen is of limited value in determining the need for an antibiotic with VRE culture-directed coverage.

Screening for vancomycin-resistant *Enterococcus* (VRE) has been utilized to identify colonized patients to prevent transmission. However, little is known on the utility of using screening to predict subsequent infection or to guide antibiotic therapy. The purpose of this study was to evaluate the predictive value of a VRE screening result for subsequent development of a VRE infection and to assess the appropriateness of antibiotic therapy in patients with a positive screening result.

Enterococci are commensal organisms of the gastrointestinal and genitourinary tract that have become important organisms in healthcare-associated infections (HAIs).^1,2^ *Enterococcus faecalis* and *E. faecium* are responsible for most human enterococcal infections including endocarditis, bacteremia, intra-abdominal infections, and urinary tract infections. Enterococci are intrinsically resistant to many antibiotics and can also acquire resistance to other antibiotics, including glycopeptides. Resistance is more common with *E. faecium* compared to *E. faecalis*.^1^ VRE has emerged as an important cause of HAIs since it was first discovered in the 1980s; it has been associated with increased morbidity, mortality and healthcare expenditure.^3–5^ Risk factors for the development of VRE infection include increased exposure to antibiotics, diabetes mellitus, hemodialysis, neutropenia, and abdominal transplantation.^6–8^ Individuals colonized with VRE are asymptomatic and may serve as a reservoir for transmission. Previous studies have shown that patients colonized with VRE have a high likelihood of developing VRE bacteremia.^5,9–11^

To reduce the transmission of resistant bacterial infections, including VRE, the Society of Healthcare Epidemiology of America (SHEA) and the Hospital Infection Control Practices Advisory Committee (HICPAC)^12,13^ have published recommendations including contact precautions for patients infected or colonized with VRE. The SHEA guidelines recommend the use of active surveillance for high-risk patients, and HICPAC emphasizes tailoring the use of active surveillance based on local conditions, prevalence, and feasibility.^12,13^ Active surveillance is conducted using a rectal or perirectal swab to identify VRE colonization. Unlike methicillin-resistant *Staphylococcus aureus* (MRSA), there are no effective methods for VRE decolonization.^13^

Although active surveillance is intended to identify colonization and prevent transmission, whether a positive VRE screen can accurately predict the development of a subsequent VRE infection remains unknown. Previous studies have demonstrated that a negative VRE screening result has a high specificity and negative predictive value (NPV) for the development of a VRE infection, but the correlation between a positive screen and subsequent VRE infection has yet to be determined.^14–16^

## Methods

In this retrospective study, we evaluated VRE screening of patients between June 1, 2015, and May 31, 2018, at a 280-bed, academic, tertiary-care hospital. Screening is routinely performed on admission for hematologic malignancy and liver transplantation patients. The outcomes of interest in this study included the appropriateness of antibiotic therapy in patients who underwent VRE screening, as well as sensitivity, specificity, PPV, and NPV of VRE screening results for confirmed VRE infection. This study was approved by the Mayo Clinic Institutional Review Board.

Patients 18 years or older who underwent screening for VRE by polymerase chain reaction assay (PCR) and had a bacterial culture from 7 days before to 90 days after the screen were included. Only the first screening result was included for patients who had multiple VRE screening tests. All bacterial cultures for patients undergoing screening were reviewed for the specified period, and patients were classified as having a VRE infection if any culture showed at least 1 *Enterococcus* isolate with vancomycin resistance and an identifiable source of infection. Patients with VRE from urine cultures were evaluated for asymptomatic bacteriuria or colonization and were excluded from the analysis.

Appropriateness of VRE-directed therapy was defined as therapy with linezolid or daptomycin for patients who had a positive VRE culture with antibiotic susceptibility and an identifiable source of infection or had no clinical improvement on alternative therapy and not solely on VRE screening alone. Patients were excluded if they received linezolid or daptomycin for a non-enterococcal infection. If appropriateness was unclear, an infectious diseases physician determined appropriateness. Antibiotic exposure for 30 days prior to and 90 days after positive VRE screen was evaluated. VRE cultures were processed by the microbiology laboratory in accordance with Clinical and Laboratory Standards Institution Guidelines. Rectal swabs were tested using the Xpert vanA assay (Cepheid, Sunnyvale, CA).

### Statistical analysis

Univariate descriptions were provided for baseline characteristics. Discrete variables were evaluated using the χ^2^ test, and the Wilcoxon rank-sum test was used for continuous variables.

For the sensitivity and specificity calculations, a positive VRE screen in a patient with VRE infection was deemed a true positive, and a negative screen in a patient without VRE infection was considered a true negative. The sensitivity, specificity, positive predictive value (PPV) and NPV of VRE screening for detecting VRE infection were calculated using SAS version 9.4 software (SAS Institute, Cary, NC).

Subgroup univariate and multivariate analyses were used to evaluate risk factors for the development of VRE infection for all patients who had at least 1 culture positive for VRE. Specifically, sex, race, number of comorbidities, days of oral vancomycin exposure, days of intravenous vancomycin exposure, and days of antipseudomonal antibiotic exposure were evaluated.

## Results

**Fig. 1.**
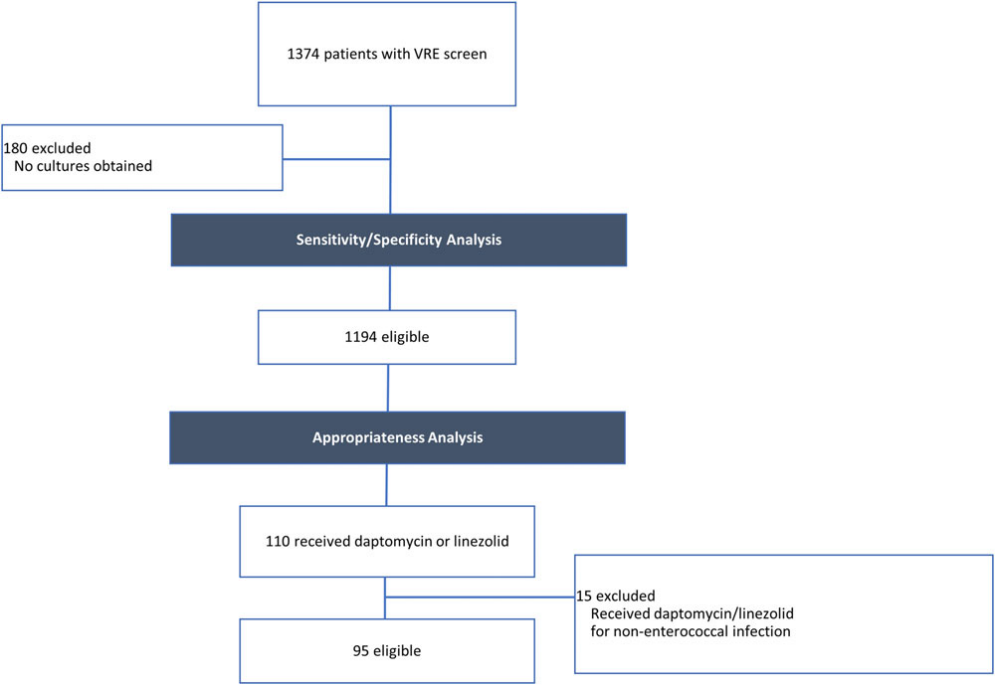
Patient eligibility. Patient eligibility for the sensitivity/specificity and appropriateness analyses.

During the study period, 1,374 patients had a VRE screen. Of the patients with a VRE screen, 1,194 patients had at least 1 bacterial culture and were included in the specificity and sensitivity analysis. Moreover, 110 patients who met the inclusion criteria received at least 1 dose of either daptomycin or linezolid. Also, 15 patients were excluded because they received daptomycin or linezolid therapy for a documented non-enterococcal infection; thus, 95 patients were included in the antibiotic appropriateness analysis. Patient eligibility is shown in Figure 1.

**Fig. 2.**
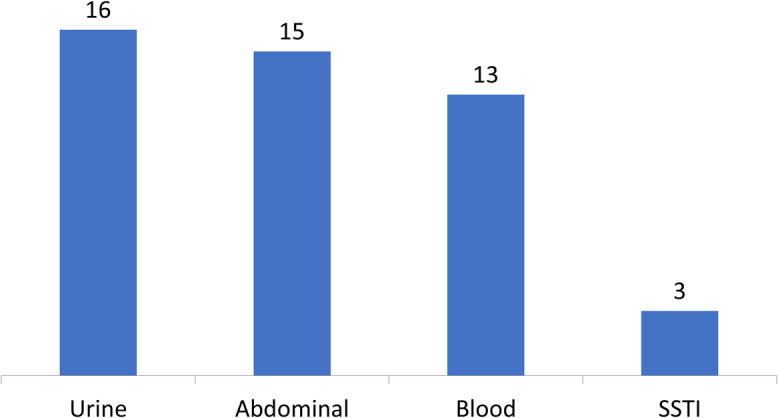
Culture sites positive for VRE. Patients may have multiple culture sites positive for VRE. Note. SSTI, skin and soft-tissue infection.

In total, 141 patients (12%) had positive VRE screening results, indicating VRE colonization. Also, 499 patients had a positive culture result, of whom 107 patients (9%) had cultures positive for *Enterococcus*. Vancomycin resistance was demonstrated in 42 patients, indicating a prevalence of 39.2% for VRE infection in the patient cohort with *Enterococcus* and 3.5% prevalence in the cohort of patients with a VRE screening test. Culture sites positive for VRE are shown in Figure 2. The average number of VRE screening tests obtained per patient was 2. In the subset of patients who were identified as true positives, the average number of days from VRE screening test to positive culture was 14.7; however, only the first screening result was included in the analysis and therefore, this number may not reflect time to positive culture.

Baseline demographic and clinical characteristics were similar in patients with a positive or negative VRE screening result (Table 1). The mean patient age was 57.8 years and 58.5% were male. Notably, patients who had a history of stem cell transplant, who were neutropenic (neutrophil count <0.5×10^9^/L), or who were febrile during the admission were more likely to have a negative VRE screening result. Patients with renal dysfunction, history of solid-organ transplant, prolonged ICU length of stay, and longer antibiotic exposure were more likely to have a positive VRE screening result.

**Table 1.** Baseline Characteristics of Patients with a Positive or Negative Vancomycin-Resistant *Enterococcus* (VRE) Screen

Characteristic	Positive VRE Screen (n = 141)	Negative VRE Screen (n = 1,053)	Total (n = 1,194)	*P* Value
Sex, no. (%)				.80
Male	81 (57.4)	617 (58.6)	698 (58.5)	
Female	60 (42.6)	436 (41.4)	496 (41.5)	
Age, mean y ± SD	59.1 ± 12.61	57.6 ± 12.99	57.8 ±12.95	.26
Weight, mean kg ± SD	78.8 ± 21.98	82.4 ± 21.38	81.9 ± 21.47	.06
BMI, mean ± SD	29.3 ± 19.63	29.1 ± 12.83	29.1 ± 13.79	.04
Race, no. (%)				.29
White	124 (87.9)	890 (84.5)	1014 (84.9)	
Other	17 (12.1)	163 (15.5)	180 (15.1)	
Comorbidities, no. (%)				
Stem cell transplant	34 (24.1)	442 (42)	476 (39.9)	<.01
Solid organ transplant^a^	54 (38.3)	319 (30.3)	373 (31.2)	.05
Renal dysfunction^b^	26 (18.4)	78 (7.4)	104 (8.7)	<.01
Antibiotic exposure, mean ± SD				
Days of antibiotic therapy,	20.6 ± 15.71	17.8 ± 16.6	18.2 ± 16.52	<.01
Days of oral vancomycin,	1.3 ± 3.82	1.0 ± 4.55	1.1 ± 4.47	<.01
Days of IV vancomycin,	3.6 ± 4.44	3.1 ± 5.02	3.2 ± 4.96	<.01
Days of antipseudomonal antibiotics,	9.7 ± 10.75	9.2 ± 11.28	9.2 ± 11.22	.25
Neutropenia, no. (%)	21 (14.9)	324 (30.8)	345 (28.9)	<.01
Febrile, no. (%)	46 (32.6)	437 (41.5)	483 (40.5)	.04
ICU length of stay, mean ± SD	7.7 ± 10.45	6.9 ± 12.42	7.1 ± 12.1	.01
Positive culture, no. (%)	89 (63.1)	409 (38.8)	498 (41.7)	<.01

Note. SD, standard deviation; BMI, body mass index; IV, intravenous; ICU, intensive care unit.

a History of solid-organ transplant includes kidney, liver, pancreas, and heart transplant. Per hospital protocol, only liver transplant patients are routinely screened on admission.

b Renal dysfunction was defined as need for renal replacement therapy, a doubling of the serum creatinine level during the index admission, or an increase in serum creatinine level to >2.0 mg/dL.

In total, 80 patients received at least 1 dose of daptomycin, and 47 patients received at least 1 dose of linezolid. In patients with a positive screening result, 27.7% received daptomycin and 20.6% received linezolid (*P* < .01). Although patients with a positive screening result were more likely to receive VRE-directed therapy, there was no difference in the appropriateness of daptomycin or linezolid between the group whose VRE screening results were positive and the group whose VRE screening results were negative (59.3 vs 61.0%; *P* = .8657). Patients with VRE infection were 13 times more likely to have had a positive VRE screening result (OR, 13.13; 95% CI, 6.89–25.04; *P* < .0001). Table 2 shows VRE colonization status in patients with at least 1 bacterial culture drawn. The sensitivity, specificity, PPV and NPV of VRE screening for VRE infection are presented in Table 3.

**Table 2.** Vancomycin-resistant *Enterococcus* (VRE) Colonization Status in Patients with Bacterial Cultures

Culture Result	No VRE Infection, No.	VRE Infection, No.
Negative VRE swab	1,036	17
Positive VRE swab	116	25
Total	1,152	42

**Table 3.** Sensitivity/Specificity Analysis of VRE Screen for VRE Infection

Test Characteristics	% (95% CI)
Sensitivity	54 (37–71)
Specificity	89 (88–91)
Positive predictive value	13 (8–20)
Negative predictive value	98 (98–99)

Note. CI, confidence interval.

Univariate analysis revealed that oral or IV vancomycin, antipseudomonal antibiotics, renal dysfunction, and history of a solid-organ transplant were risk factors for VRE infection. Patients with a history of stem cell transplant were less likely to develop VRE infection in our cohort. A multivariate analysis demonstrated that patients were more likely to develop VRE infection if they received antipseudomonal antibiotics (*P* < .0001) or had multiple comorbidities (*P* = .0496).

## Discussion

The benefits of utilizing active VRE surveillance to prevent transmission are known.^17^ A multicenter retrospective review concluded that routine VRE surveillance on admission followed by weekly screening facilitated the identification of VRE carriers in high-risk medical units.^18^A prospective, observational study in ICU patients concluded that twice-weekly VRE screening can effectively identify colonized patients and reduce transmission.^19^

However, little is known regarding the benefits of VRE screening to predict or rule out VRE infection. Most patients who undergo VRE surveillance do not develop an enterococcal infection. Previous studies have reported that 12%–25% of patients with a positive screen on admission develop a VRE infection during the hospitalization.^7,11,18^ A study of 1,666 patients screened for VRE in a hematology-oncology unit found that 9.7% of patients were colonized and that 12.4% of colonized patients developed a VRE bloodstream infection.^20^ A prospective review of medical ICU patients reported that 17.6% of patients were colonized with VRE on ICU admission; of these, 11.9% subsequently developed a VRE infection during the hospitalization.^7^

In our study, 9% of patients developed an enterococcal infection, of whom 39% were due to VRE, amounting to 3.5% of all patients screened for VRE. Among patients with a positive VRE screening result, the likelihood of VRE infection was 13 times higher than in patients with a negative VRE screening results. This finding may support empiric therapy for VRE infection in patients with a positive VRE screen and reduce delay of appropriate antibiotic therapy, which can lead to increased mortality.^21^

In this cohort, the PPV of a VRE screen was low at 13%. Thus, although the risk for VRE infection is higher with a positive screen, the likelihood of developing VRE infection based on the screen is low. Continuation of empiric VRE coverage should be reconsidered in the absence of cultures positive for VRE or other indication for VRE-active antibiotics. On the other hand, the specificity and NPV were high at 89% and 98%, respectively; suggesting that a patient with a negative VRE screen is less likely to have a VRE infection and does not require empiric antibiotic coverage for VRE.

A retrospective review of patients with enterococcal infections who had undergone VRE screening found a specificity of 97% and a sensitivity of 70% for sterile-site VRE infection.^15^ Another study evaluated patients with enterococcal bloodstream infections who were routinely screened for VRE on admission and stratified PPV and NPV based on the proportion of VRE. This study reported a PPV of >50% for VRE proportion >25% and NPV of 90% and 95% for a VRE proportion <27% and <15%, respectively.^16^ A prospective study of ICU patients found that twice-weekly screening had a sensitivity of 89%, specificity of 90%, PPV of 44%, and NPV of 99%.^19^ Univariate and multivariate analyses demonstrated that patients in our cohort who received intravenous or oral vancomycin or antipseudomonal antibiotics, had renal dysfunction, or with a history of solid-organ transplantation were more likely to develop VRE infection.

Collectively, these results suggest consideration of a positive screen along with patient-specific clinical factors to determine the need for empiric antibiotic therapy for VRE. In addition, empiric therapy for VRE may not be necessary for a patient with a negative VRE screen in the absence of risk factors for VRE infection. In this study, patients with a positive VRE screen were more likely to have received broad-spectrum antibiotics and were also more likely to have received daptomycin or linezolid. These results demonstrate the importance of antimicrobial stewardship interventions to optimize use of broad-spectrum antibiotics and reduce VRE infections.

The strengths of this study include a large sample size of patients with VRE screening on admission. The patients screened were considered to have a higher risk for VRE infection based on established risk factors and in accordance with established infection prevention and control guidelines. This study also evaluated the impact of a VRE screen result on VRE-directed therapy and is the first known study, to our knowledge, to evaluate the impact of antibiotic appropriateness in relation to VRE screening.

This study had several limitations. It had a single-center, retrospective design, and a small percentage of screened patients had VRE infection. Only the first VRE screen was included in the study; a different screening result may change our findings. Finally, no data on patient outcomes were collected.

In conclusion, VRE screening did not affect the appropriateness of VRE-directed antibiotic therapy. The high NPV and high specificity of the VRE screen suggest that patients with a negative VRE screen may not require empiric antibiotic therapy for VRE. Although VRE screening facilitates detection of VRE colonization and thereby reduces transmission when coupled with infection prevention measures, a positive VRE screen is of limited value in determining the need for empiric VRE antibiotic therapy. A VRE screen could be a useful decision-making tool while waiting for culture results in patients with risk factors for enterococcal infections. Further research is needed to determine optimal utilization of VRE screening for prediction and empiric treatment of VRE infections.
